# Retraction Note: Dramatic HIV DNA degradation associated with spontaneous HIV suppression and disease-free outcome in a young seropositive woman following her infection

**DOI:** 10.1038/s41598-024-60734-y

**Published:** 2024-05-01

**Authors:** Philippe Colson, Catherine Dhiver, Catherine Tamalet, Jeremy Delerce, Olga O. Glazunova, Maxime Gaudin, Anthony Levasseur, Didier Raoult

**Affiliations:** 1 IHU Méditerranée Infection, 19-21 boulevard Jean Moulin, 13005 Marseille, France; 2 Aix-Marseille Univ., IRD, AP-HM, MEPHI, 19-21 boulevard Jean Moulin, 13005 Marseille, France

Retraction of: *Scientific Reports* 10.1038/s41598-020-58969-6, published online 13 February 2020

Editors have retracted this Article.

After publication of this Article concerns about consent to participate were brought to the attention of the Editors. While the Articles states that written informed consent was obtained from the patients, the Authors were not able to provide documentation confirming that the patients who provided the samples used in this study gave consent for these samples to be used for research purposes.

All Authors disagree with the retraction.

